# Enhanced phytoremediation of cadmium-contaminated soil using chelating agents and plant growth regulators: effect and mechanism

**DOI:** 10.1098/rsos.240672

**Published:** 2024-09-25

**Authors:** Qiao Yang, Hao Yu, Chen Yang, Zhongqiu Zhao, Zhengshan Ju, Jinman Wang, Zhongke Bai

**Affiliations:** ^1^ Land Consolidation and Rehabilitation Center, Ministry of Natural Resources, Beijing 100035, People’s Republic of China; ^2^ School of Land Science and Technology, China University of Geosciences (Beijing), Beijing 100083, People’s Republic of China; ^3^ Technology Innovation Center of Land Engineering, Ministry of Natural Resources, Beijing 100035, People’s Republic of China; ^4^ College of Resource and Environment, Shanxi Agricultural University, Taigu 030801, People’s Republic of China

**Keywords:** chelating agents, plant growth regulator, microbial communities, plant extraction

## Abstract

The heavy metal cadmium (Cd) is a major threat to food safety and human health. Phytoremediation is the most widely used remediation technology, and how to improve the remediation efficiency of phytoremediation has become a key issue. In this study, we constructed an intensive phytoremediation technology for remediation of Cd-contaminated soil with biodegradable chelating agent and plant growth regulator combined with maize and investigated the mechanism of this technology. The results showed that the best remediation effect was achieved in the treatment with 10^−6^ mol l^−1^ gibberellic acid (GA_3_) and 6 mmol kg^−1^ aspartate diethoxysuccinic acid (AES) combined with maize. In this treatment, the total biomass and extraction efficiency of maize were 3.6 and 8.67 times higher than those of the control, respectively, and the antioxidant enzyme activities of maize were also increased. The soil was enriched with dominant bacterial genera that promote plant growth and metabolism and tolerance to heavy metal stress, which in turn promoted maize growth and Cd accumulation. Structural equation modelling results indicated a large effect of plant Cd concentration and plant antioxidant enzyme activity on plant Cd extraction. The enhanced phytoremediation technology showed good potential for safe use of Cd-contaminated soil.

## 1. Introduction

With the accelerated development of industry, agriculture and mining, farmland soil polluted with heavy metals has become an increasingly serious environmental problem [1,2]. Cadmium (Cd) is a non-essential nutrient for living organisms and is listed as a heavy metal pollutant because of its strong migration and transformation characteristics in the environment and high hazard to human beings [3]. Elemental Cd in soil can adversely affect plant growth and may also be toxic, mutagenic and carcinogenic to these organisms at low concentrations [4,5]. Cd in agricultural soil is absorbed by plant roots and then transferred to the ground, which threatens human health through the food chain [6]. Therefore, there is an urgent need for effective remediation technology to reduce heavy metal pollution in soil.

Common methods for the remediation of heavy metal-contaminated soil include physical, chemical and biological techniques [7,8]. Physical and chemical remediation techniques include mulching, soil drenching and electric remediation, which are often costly and have a negative impact on the original soil structure and ecosystem [9,10]. Conversely, phytoremediation is an environmentally friendly technology for remediating heavy metal pollution in soil [11]. Using plants to extract heavy metals is cheaper, less invasive to the soil and ecology, and it is an easily operated *in situ* remediation technology [12,13]. However, there are two major factors limiting the efficiency of phytoremediation: the biological effectiveness of heavy metals in the soil and the tolerance of plants to contaminated soil, i.e. plant biomass [14]. In our previous study, we found two biodegradable chelating agents and their ability to effectively promote the extraction of Cd from maize at optimal concentrations (3 mmol kg^−1^ GLDA (glutamic acid *N*,*N*-diacetic acid tetrasodium salt) and 6 mmol kg^−1^ aspartate diethoxysuccinic acid (AES)) with the advantage of strong chelating ability, but the remediation efficiency of heavy metals by plants is affected by plant biomass and soil heavy metal effectiveness [15,16]. Therefore, finding a remediation technology that can enhance the biomass and Cd tolerance of maize is necessary for removing Cd from soils and is important for achieving safe use of Cd-contaminated agricultural land.

In addition, some studies have shown that the application of plant growth regulators (gibberellic acid, GA_3_) can fix heavy metal ions in plants and improve plant resistance by regulating physiological and biochemical processes, thereby promoting plant growth [17]. The plant growth regulator gibberellin can reduce oxidative stress caused by heavy metals. It can also significantly improve the uptake of heavy metals by maize and increase the total chlorophyll concentration in plants [18,19]. Compared with an auxin or a cytokinin, gibberellin has become the preferred hormone for assisting plants to repair heavy metal pollution due to its unique advantages in promoting plant growth, improving antioxidant capacity, and relieving heavy metal stress [20]. However, many of the current studies on intensive phytoremediation of Cd-contaminated soil only use a single chelating agent or a single plant growth regulator, and the effect of the combination of the two on intensive phytoremediation of Cd-contaminated soil is still unclear.

Microorganisms are an important part of the soil, and they are the basis for the healthy operation of the soil ecosystem [21,22]. Human disturbance affects the activity of soil microorganisms. The growth and activity of soil microorganisms are very sensitive to changes in soil structure and physical and chemical properties [23]. Anthropogenic disturbances not only affect the activity of soil microorganisms but also further affect the metabolic activity and community succession of soil microorganisms. However, there have been few studies on the effects of these artificially added compounds on soil microorganisms. Therefore, it is necessary to investigate the activities and structural changes in soil microorganisms during phytoremediation of heavy metal pollution.

The study of heavy metal-contaminated soils remediated by heavy metal-accumulating plants with large biomass has become a new research idea to replace the hyper-accumulating plants. Maize has the advantage of rapid growth and high biomass to compensate for the small biomass and slow growth of super-enriched plants for Cd. It has been shown that maize is a tolerant plant for remediation of heavy metal-contaminated soil with strong enrichment and translocation capacity of Cd [24,25], which affords better results in remediation of Cd contamination in soil, and can be used as a phytoremediation material for the extraction of Cd from contaminated soil [26]. In addition, it has been shown that the root system of maize develops faster at the seedling stage and has a high enrichment capacity for Cd, and the enrichment of heavy metals at the seedling stage will affect the growth and development at the later stage, such as affecting the pulling stage, the ear stage and the flower and grain stage of maize, and the seriousness of which will directly lead to the death of maize at the pulling stage [27]. Therefore, maize was selected as the phytoremediation material in this study to carry out experimental studies at the seedling stage.

Our previous study found that biodegradable chelating agents AES and GLDA could effectively promote the extraction of Cd from maize [28]. However, the efficiency of plant remediation of heavy metals was affected by plant biomass and soil heavy metal effectiveness. The application of plant growth regulators can promote the growth and development of restoration plants, enhance the enrichment of heavy metals by plants, improve the resistance of plants and then improve the efficiency of phytoremediation. In order to investigate the effect of chelating agent combined with plant growth regulator to enhance the remediation of Cd-contaminated soil by maize, this study will use the selected biodegradable chelating agent combined with plant growth regulator GA_3_ to enhance the remediation of Cd-contaminated soil on agricultural land by maize. On the one hand, the effects of different concentrations of plant growth regulators and biodegradable chelating agents on maize growth, physiological tolerance and remediation effects will be investigated. On the other hand, the effects of intensive phytoremediation on the composition and structure of soil microbial communities were analysed to further elucidate the mechanism of this remediation technology. This study is of great significance to achieve the safe use of Cd-contaminated agricultural land.

## 2. Material and methods

### 2.1. Material preparation

The seeds of maize (*Zea mays* L.) were provided by the Henan Academy of Agricultural Sciences. GA_3_ and GLDA were purchased from Shanghai Maclean Biochemical Technology Co. Ltd. AES was purchased from Kemira Company, Finland. Topsoil samples (0–20 cm) with a medium loamy texture (31.21% sand, 30.37% clay, and 38.43% silt) were collected from the upper 0–20 cm layer of a maize field in Shunyi District, Beijing (40°6′3.87″ N, 116°53′28.80″ E), and passed through a 20-mesh sieve prior to analysis of the basic soil properties. The soil type collected is sandy loam soil. The background concentration of Cd in the original soil was 0.35 mg kg^−1^. Exogenous heavy metal Cd (in the form of CdCl_2_ solution) was added to the air-dried soil. The Cd content reached more than the risk intervention value for agricultural soil contamination stated in the ‘Soil environmental quality agricultural land soil pollution risk control standard’ (GB15618-2018). The risk intervention value for Cd content in soil is 4 mg kg^−1^ when soil pH > 7.5. After 60 days, basal fertilizer (N: 100 mg kg^−1^ soil, P: 80 mg kg^−1^ soil, K: 100 mg kg^−1^ soil) was applied in the form of urea (CO(NH_2_)_2_) and potassium dihydrogen phosphate (KH_2_PO_4_) solution. The treated soil was thoroughly mixed to maintain a moisture content at about 60% of the field moisture content and then left for one week. The basic physicochemical properties of the soil were as follows: 13.4 g kg^−1^ organic matter, 0.111% total nitrogen (TN), 0.135% total phosphorus (TP), 2.16% total potassium (TK), 103 mg kg^−1^ hydrolyzed nitrogen, 176 mg kg^−1^ effective phosphorus, 6.0 cmol kg^−1^ cation exchange, 6.53 mg kg^−1^ total Cd and pH 7.95.

### 2.2. Experimental design

The whole two-stage experiment was done in an artificial climate chamber (MGC-450HP). The instrument was purchased from Shanghai Yiheng Scientific Instrument Co. The experimental parameters of the artificial climatic chamber were set for a total of 13 h during the daytime at a temperature of 26℃ and a light intensity of 3000 lux. The experimental parameters of the artificial climatic chamber at night were set to a total of 11 h at a temperature of 18℃ with no light intensity. The soil that was treated as described above was placed in pots (500 g of soil each). The pots used for the experiment were 11 cm high, 12 cm in diameter at the top and 8 cm at the bottom. Maize seeds were tested for germination and then moved into pots containing contaminated soil. After interplanting, two plants per pot were left to perform the maize seedling tests.

Based on the results of a previous experiment [28], the concentration of chelating agent selected in this study was 3 mmol kg^−1^ GLDA and 6 mmol kg^−1^ AES. The chelating agent was applied to the soil at the 30th day of maize growth. After 24 h of chelating agent treatment, three different concentrations (10^−6^, 10^−5^ and 10^−4^ mol l^−1^) of GA_3_ were sprayed on maize leaves using a spray can. A treatment without chelating agent and plant hormone was also used as a control. A total of 12 treatments were designed for the whole experiment (table 1), and each treatment was repeated three times for the experiment.

**Table 1.** Experimental treatments.

treatment	concentration of GA_3_ (mol l^−1^)			
	0	10^−6^	10^−5^	10^−4^
0	CK	G1	G2	G3
6 mmol kg^−1^ AES	AES	AG1	AG2	AG3
3 mmol kg^−1^ GLDA	GLDA	GG1	GG2	GG3

Individual treatments and abbreviations in the table are as follows: CK (no GA_3_ and no chelating agent), AES (6 mmol kg^−1^ AES), GLDA (3 mmol kg^−1^ GLDA), G1 (10^−6^mol l^−1^ GA_3_), G2 (10^−5^mol l^−1^ GA_3_), G3 (10^−4^mol l^−1^ GA_3_), AG1 (6 mmol kg^−1^ AES + 10^−6^ mol l^−1^ GA_3_), AG2 (6 mmol kg^−1^ AES + 10^−5^ mol l^−1^ GA_3_), AG3 (6 mmol kg^−1^ AES + 10^−4^ mol l^−1^ GA_3_), GG1 (3 mmol kg^−1^ AES + 10^−6^ mol l^−1^ GA_3_), GG2 (3 mmol kg^−1^ AES + 10^−5^ mol l^−1^ GA_3_), GG3 (3 mmol kg^−1^ AES + 10^−4^ mol l^−1^ GA_3_).

It has been shown that chelating agents are effective 3−10 days after application. Therefore, in this study, plant samples were harvested after 7 days of chelator treatment and soil samples were also collected for analytical tests [29]. The whole experiment consisted of 12 treatments, with each treatment performed in triplicate.

### 2.3. Sample analysis

#### 2.3.1. Plants

During the growth cycle of maize, chlorophyll content of maize leaves was determined using a hand-held portable SPAD meter; plant height of maize was determined using a straight edge. At the end of the experiment, maize samples were collected, large pieces of soil were removed from the roots, and the surface was rinsed clean with deionized water and then drained of water. The maize was divided into above-ground part (shoot) and under-ground part (root) and cut with ceramic scissors to obtain samples from each part. Small proportions of the shoot samples were used for the determination of physiological and biochemical indices. The rest of the samples were weighed and dried, first killed at 105°C for 30 min and then dried at 70°C until constant weight. Finally, the dry weights of the shoot and root of the maize were measured.

After grinding the plant samples, microwave digestion was carried out with mixed acid (HNO_3_ : HCIO_4_ = 5 : 1 (v/v)). After filtration, the Cd content in plant was determined by ICP-MS (Agilent 7500). Celery was used for quality control, and blank test was conducted in the whole process.

The physiological and biochemical activities of the above-ground plant parts were studied following previous methods [30,31]. The malondialdehyde (MDA) content in the plants was measured by the thiobarbituric acid colorimetry method. Three grams of leaves were cut into pieces and added to 30 ml of phosphoric acid buffer. The leaves were ground into a homogenate, centrifuged, extracted and combined with the supernatant to a fixed volume, and then the enzyme solution was prepared for refrigeration. Superoxide dismutase (SOD) activity was determined by the nitrogen blue tetrazolium (NBT) method. A sample of 0.5 ml of the above enzymatic liquor was collected, and then 1.5 ml of 0.05 mol l^−1^ phosphate buffer, 0.3 ml of 130 mmol l^−1^ Met solution, 0.3 ml of 0.75 mmol l^−1^ NBT solution, 0.3 ml of 0.1 mmol l^−1^ EDTA-Na_2_ solution and 0.3 ml of 0.02 mmol l^−1^ riboflavin were added. Using a dark tube as a blank control, UV absorbance was measured at 560 nm. The activity of catalase (CAT) was determined by ultraviolet absorption. A sample of the enzyme in the amount of 2.5 ml was added to 2.5 ml of 0.1 mol l^−1^ H_2_O_2_. Afterwards, 2.5 ml 10% H_2_SO_4_ was added to the sample, which was then heated in a 30℃ water bath for 10 min, and then this solution was titrated with 0.1 mol l^−1^ KMnO_4_ until the pink coloration disappeared. The UV absorbance was measured at a wavelength of 240 nm. The activity of peroxidase (POD) was determined by the guaiacol method. A sample of the above enzyme solution in the amount of 0.1 ml was taken, and then 2.9 ml of 0.05 mol l^−1^ phosphate buffer, 1.0 ml of 2% H_2_O_2_ and 1.0 ml of 0.05 mol l^−1^ guaiacol were added. The solution was heated in a 37℃ water bath for 15 min, and then 2.0 ml of 20% trichloroacetic acid was added to terminate the reaction. The solution was then centrifuged, and the absorbance was measured at a wavelength of 470 nm.

#### 2.3.2. Soil

The physical and chemical properties of the soil were determined following the ‘Analytical methods of soil agricultural chemistry’ [32] and ‘Soil agrochemical analysis’ [33]. The pH was measured in a 1 : 2.5 ratio of soil in a water suspension by a glass electrode pH meter. The TN content was measured by semi-micro Kjeldahl method. The TP content was measured by vanadium molybdenum yellow spectrophotometry. The TK content was measured by the flame photometric method. The alkaline solution diffusion method was used for the determination of soil hydrolysed nitrogen, and the extraction-molybdenum anti-colorimetric method was used to determine available phosphorus. The determination of soil cation exchange capacity was conducted using the ammonium chloride–ammonium acetate exchange method. Soil organic matter was determined using a TOC analyser.

Soils were microwave digested using mixed acid HCl : HNO_3_ : H_2_O_2_ = 3 : 1 : 1 (v/v/v), after acid drive, volume determination and filtration, the Cd content in soil was determined by ICP-MS (Agilent 7500). The available Cd was measured by the CaCl_2_-DTPA leaching method, and the Cd concentration was measured with an atomic spectrophotometer. Parallel samples, blank samples and national standard samples (GBW07405a (GSS-5a)) were set up for the quality control of the determination process, and the standard deviations of the parallel samples were within 5%, and the recoveries of each heavy metal element ranged from 86.3% to 113.3%.

#### 2.3.3. Soil microbial analysis

After the experiment, 2 g of fresh soil samples was collected and frozen at −80℃ for soil bacterial community structure determination. The rest of the soil was naturally air-dried and set aside.

Total DNA was extracted from microbial communities according to the instructions of the EZNA^®^ soil DNA kit (Omega Bio-tek, Norcross, GA, USA). PCR amplification of the V3–V4 hypervariable region of the 16S rRNA gene was performed using the primers 338F (5′-ACTCCTACGGGAGGCAGCAG-3′) and 806R (5′-GGACTACHVGGGTWTCTAAT-3′). The amplification procedure was as follows: 95℃ pre-denaturation for 3 min; 27 cycles of denaturation at 95℃ for 30 s, annealing at 55℃ for 30 s and extension at 72℃ for 45 s; followed by stable extension at 72℃. PCR products from the same sample were mixed and recovered from a 2% agarose gel. The recovered products were purified using an AxyPrep DNA Gel Extraction Kit (Axygen Biosciences, Union City, CA, USA) and quantified using a Quantus™ fluorometer (Promega, USA). The purified amplification products were pooled in equimolar concentrations and subjected to pair end sequencing on the Illumina-MiSeq platform according to the standard protocol. The 16S rRNA gene fragment amplification and Illumina MiSeq sequencing were performed at Shanghai Meiji Biomedical Technology Co., Ltd.

The operational taxonomic units (OTUs) were clustered using UPARSE at 97% similarity. Chimeric sequences were identified and removed using UCHIME. Each 16S rRNA gene sequence was categorized and analysed against the SILVA (SSU115) 16S rRNA database at a 70% confidence level.

### 2.4. Data processing and analysis

The plant extraction efficiency of Cd was determined as follows: Cd concentration of shoot × dry weight of above-ground plant/total Cd concentration in soil.

The analysis of variance, correlation, standard deviation and significance of differences in the data were performed using SPSS 25.0 software. Canoco 5.0, which is a software for ranking analysis of multivariate data in related fields, was used to perform redundancy analysis (RDA) for the relationship between soil bacterial abundance and soil physicochemical properties. Correlation heat maps can conveniently show the correlation between multiple variables. In this article, we used the Correlation Plot App in Origin to perform Spearman analysis and made correlation heat map of the soil microbial community structure and soil physicochemical properties. The structural equation model (SEM) was constructed using Amos 22.0 software for soil physicochemical properties and the soil microbial community diversity and plant growth.

## 3. Results and analysis

### 3.1. Biomass and physiological and biochemical activities of maize under different treatments

**Figure 1.**
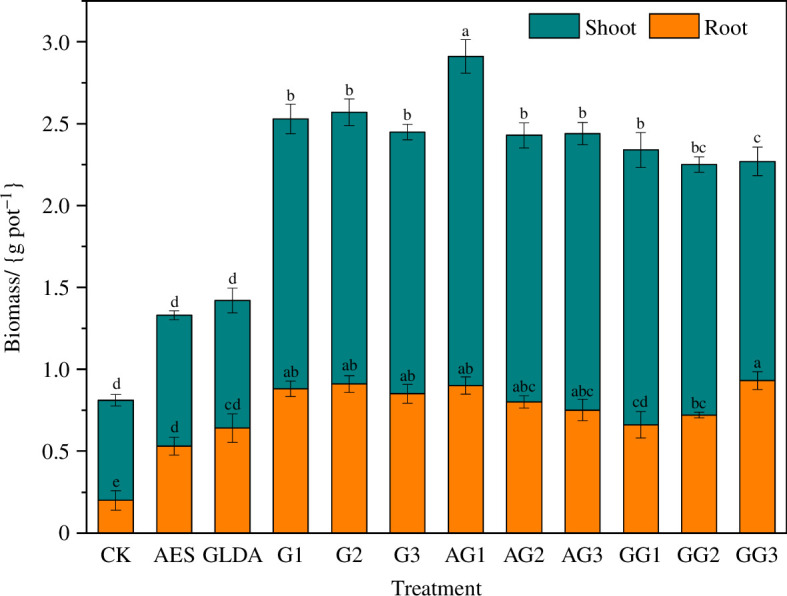
Effect of GA_3_ and chelator application on maize biomass. Note: different lowercase letters indicate significant differences between treatments (*p <* 0.05).

All of the maize showed good growth during the experiment and no typical phytotoxic symptoms, such as leaf necrosis or branch wilting, were observed. The shoot and root biomass of maize under different treatments are shown in figure 1. All treatments significantly increased the maize biomass compared to CK (*p <* 0.05). The chelator combined with GA_3_ treatment and GA_3_ treatment alone had a comparable effect and both were more effective than the chelator treatment. Under the GA_3_ addition treatment alone, the above-ground biomass, root biomass and total biomass were ordered as G2 > G1 > G3, which were 1.66, 0.91 and 2.57 g pot^−1^, respectively, under the G2 treatment, which was an increase of 172.13%, 355% and 217.28%, respectively, compared with the control. In the right concentration range of GA_3_, the biomass increases with increasing GA_3_ concentrations. However, when the concentration is too high, the maize biomass decreases [34,35].

In addition, for the different chelating agent treatments, the results of the study showed that the plant biomass was lower in the GLDA treatment than in the AES treatment at the same concentration of plant growth regulator treatment. Under the combined GA_3_ treatment with chelating agents, both showed higher biomass under the low GA_3_ concentration treatment (AG1 and GG1 treatment). The highest maize biomass was achieved under the AG1 treatment, with an above-ground biomass of 2.01 g pot^−1^, root biomass of 0.9 g pot^−1^ and total biomass of 2.91 g pot^−1^, which were 229.51%, 350% and 259.26% over control, respectively.

Soil Cd stress affects the production and scavenging of reactive oxygen radicals (ROS) in plants. The accumulation of ROS leads to membrane lipid peroxidation, resulting in MDA expression as the main product, which serves an important indicator. Plants possess a powerful defence system. When under stress, plants can mitigate heavy metal toxicity by regulating antioxidant enzyme activity and scavenging ROS to relieve heavy metal stress. The main antioxidant enzymes include SOD, CAT and POD.

**Figure 2.**
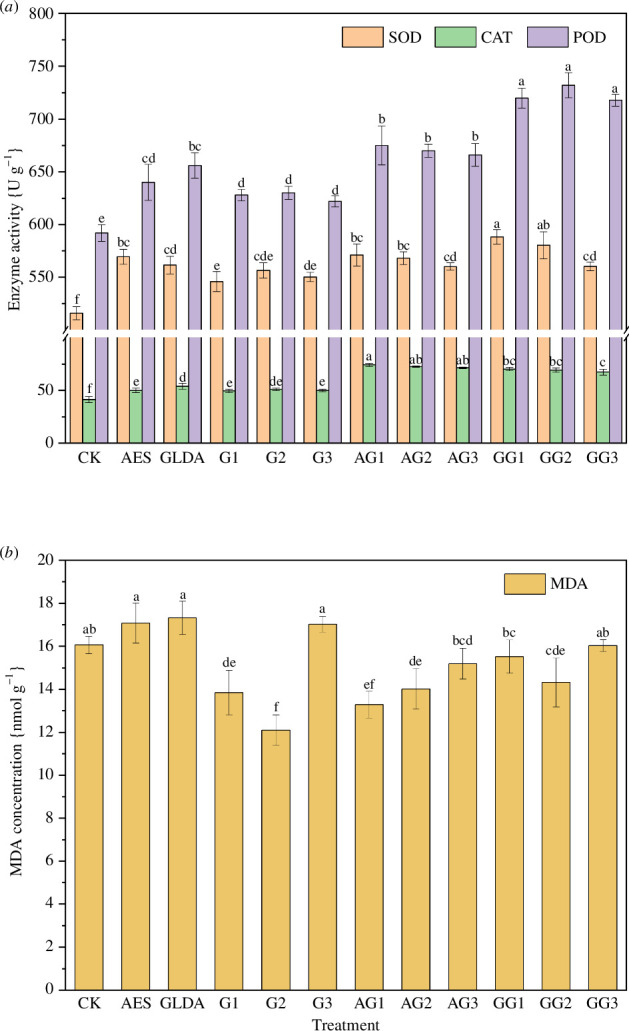
Changes in antioxidant enzyme activity (*a*) and MDA content (*b*) in maize under different treatments.

The antioxidant enzyme activities and MDA contents in maize under different treatments are shown in figure 2*a,b*. All treatments significantly increased the SOD, CAT and POD antioxidant enzyme activities when compared to CK (*p <* 0.05). The changes in MDA content under different treatments are shown in figure 2*b*. Compared with CK, the SOD activity of maize leaves in the chelator AES and GLDA treated groups increased by 10.39% and 8.84%, respectively; CAT activity increased by 20.73% and 30.12%, respectively; and POD activity increased by 8.11% and 10.81%, respectively. The MDA content also increased by 6.33% and 7.85%, respectively, compared with CK.

The single GA_3_ treatment also increased antioxidant enzyme activity in the maize, weakening the extent of cell membrane damage. The results of this study show that the SOD, CAT and POD activities increased by 5.81–7.87%, 19.56–23.33% and 5.07–6.42% under GA_3_ treatment alone, respectively. The increasing concentrations of GA_3_ all showed an increasing trend followed by a decreasing trend. MDA was the lowest under G2 treatment, with a 24.65% reduction compared to CK, and increased at the high concentration treatment (G3).

Figure 2*a* shows that the chelator combined with GA_3_ treatment further increased the antioxidant enzyme activity in the maize. Both SOD and CAT had the highest activity under the GA_3_ low phytohormone treatment, and their activity decreased with the increasing phytohormone concentration. The POD activity was highest in the AG1 and GG2 treatment groups. The MDA content decreased the most (by 17.26%) in the AG1 treatment and less in the medium and high concentrations. The GG2 treatment decreased by 10.83% compared to CK.

### 3.2. Extraction of Cd from maize under different treatments

**Figure 3.**
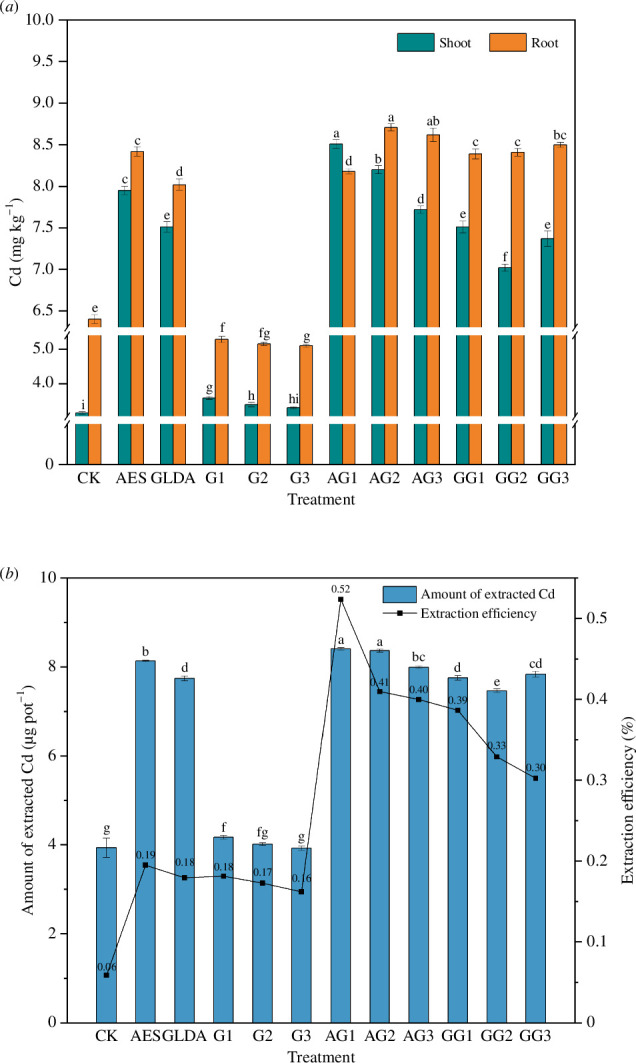
Concentration (*a*) and extraction (*b*) of Cd in maize under different treatments.

The Cd contents in the shoots and roots of maize under different treatments are shown in figure 3*a*. Compared with CK, the Cd concentrations under the chelator addition treatment alone and the chelator combined with GA_3_ treatment were comparable, and both were significantly greater than those under GA_3_ addition alone. The total Cd extraction and extraction efficiency of maize under different treatments are shown in figure 3*b*. Compared with CK, the extractions under the chelator addition alone and GA_3_ addition alone treatments were comparable, and both were lower than the extraction of Cd by maize under the synergistic treatment.

The above-ground Cd concentration in all treatments showed an increasing trend compared to CK. Additionally, the Cd concentration in roots increased significantly compared with the control under the chelator alone and chelator combined with GA_3_ treatment, but decreased significantly under the GA_3_ alone treatment (*p <* 0.05).

Under the GA_3_ treatment alone, the Cd concentration in the shoots increased by 4.76–13.65% and decreased in the roots by 17.5–20.31% compared to CK. The Cd concentration in the shoots was 3.58 mg kg^−1^ for the G1 treatment, which was 13.65% higher than that for the control. The highest Cd concentration in the roots was 5.28 mg kg^−1^, which was 17.5% less than the control.

Under the chelator combined with GA_3_ treatment, the Cd concentration increased by 122.86–170.16% in the shoots and 27.81–36.09% in the roots, compared with CK. Under the same concentration of GA_3_ treatment, above-ground and root Cd concentrations were significantly lower in the GLDA treatment than in the AES treatment (*p <* 0.05). Under AG1 treatment, the Cd concentration in the shoots peaked at 8.51 mg kg^−1^, which was 2.7 times higher than that of the control. Under AG2 treatment, the Cd concentration in the roots peaked at 8.71 mg kg^−1^, which was 1.36 times higher than that of the control. The total amount of Cd extracted by maize under AG1 treatment was maximum at 8.41 μg g^−1^ DW with an extraction efficiency of 0.52% (there is a visual representation in figure 3*b*). The magnitude of the plant extraction efficiency was as follows: AG1 > AG2 > AG3 > GG1 > GG2 > GG3 > AES > G1 > GLDA > G2 > G3 > CK. The extraction efficiency of soil heavy metals by plants determines the phytoremediation effect of soil heavy metal pollution.

### 3.3. Diversity indices of soil microbial communities under different treatments

Soil microorganisms play an important role in the nutrient cycle of the soil ecosystem. They can also be used as a sensitive indicator of the state of the soil ecosystem during soil heavy metal restoration [36,37]. After chelation treatment, six treatments, namely CK, AES, GLDA, G1, AG1 and GG1, were selected for high-throughput sequencing analysis of the 16S rRNA gene based on the above analysis results.

**Figure 4.**
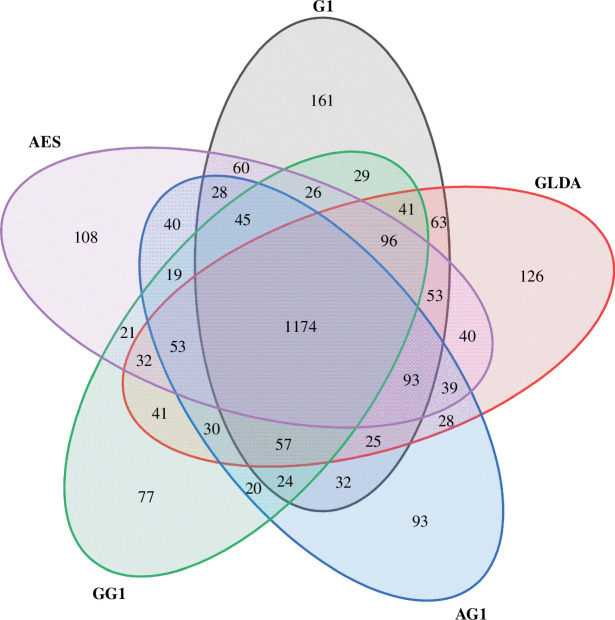
A Venn diagram of soil microbial community under different treatments. Note: different colours in the figure represent different treatments, overlapping numbers represent the number of species shared by multiple groups and non-overlapping numbers represent the number of endemic species.

Table 2 provides the abundance-based coverage estimator (ACE), Chao, Shannon and Sobs diversity indices of the soil samples under the different treatments. The coverage rate of all sequenced samples was higher than 99.7%, indicating that the results reflect the true status of the microorganisms in the samples. The Sobs index represents the number of observed OTUs. Chao and ACE indices are used to reflect the changes in the abundance of the community. The higher the index, the higher the abundance of the community [38]. The Shannon index mainly represents the microbial community diversity and homogeneity. The greater the Shannon index, the higher the community diversity [39]. The number of OTUs in the soil microbial community under the six treatments was as follows: CK > G1 > GLDA > AES > GG1 > AG1. All treatments reduced the diversity of microbial communities in the soil as compared to CK, while the community diversity index was lower under the combined treatments, i.e. AG1 and GG1 treatments. A Venn diagram (figure 4) shows the number of common and unique OTUs in each treatment. Overall, 1174 common OTUs were detected in the samples from the different treatment groups, representing 58.50%, 58.97%, 60.92%, 65.22% and 65.77% of the total OTUs in the G1, GLDA, AES, AG1 and GG1 treatments, respectively. The specific Cd-contaminated environment may be the main reason for the shared OTUs.

**Table 2.** Diversity indices of soil microbial communities under different treatments.

treatment	Shannon	ACE	Chao	Sobs	coverage
CK	4.91	651.26	649.76	610	99.97%
G1	4.89	645.87	646.78	601	99.83%
AES	4.69	645.18	658.78	582	99.81%
GLDA	4.77	632.50	628.07	584	99.86%
AG1	4.18	619.03	625.02	562	99.83%
GG1	4.62	635.75	631.80	568	99.78%

Similar to the Sobs index, lower values of Chao1 and ACE were observed in the soil microbial community of the AG1 treatment (table 2). This indicates that the abundance of the microbial community in the soil was reduced under the combined treatment.

The Shannon indices of the soil community from the six treatments were as follows: CK (4.91) > G1 (4.89) > GLDA (4.77) > AES (4.69) > GG1 (4.62) > AG1 (4.18). This shows that all treatments decreased the abundance and diversity of the soil bacterial community compared to CK.

### 3.4. Soil bacterial community structure under different treatments

**Figure 5.**
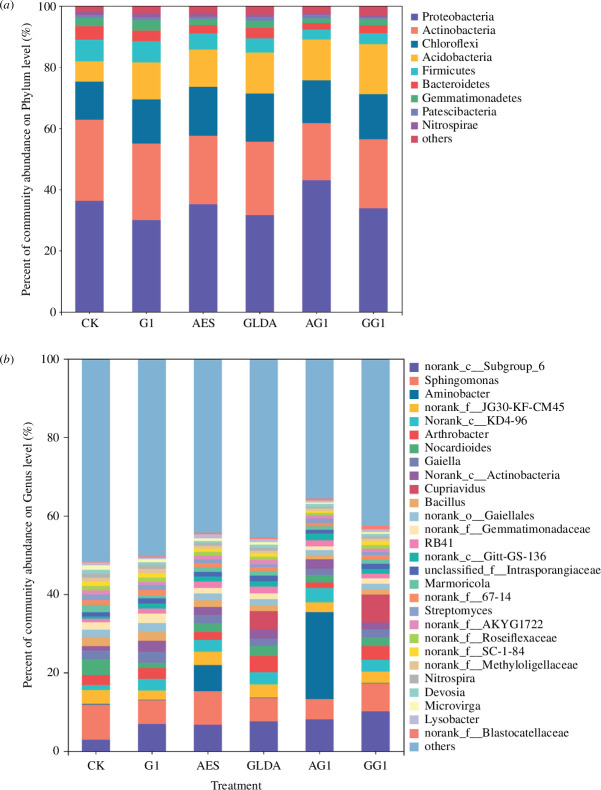
Microbial community structure at the phylum level (*a*) and genus level (*b*) in soil samples under different treatments.

In order to study the differences in the bacterial community structure of the soil samples before and after the treatment, statistical analysis was performed on all samples at the phylum level. Figure 5*a* shows the structure of soil microbial communities at the phylum level for the different treatments. It can be seen that the main constituent phyla are similar between the different treatments, but there are large differences between their relative abundances. The dominant phyla observed in this study include Proteobacteria, Actinobacteria, Chloroflexi, Acidobacteria, Firmicutes and Bacteroidetes.

Among all the treatments, Proteobacteria had the highest relative abundance. Compared to CK (36.25%), the abundance in the other treatments decreased, except for AG1 (43.06%), which increased. The Proteobacteria abundance of the other four treatments was as follows: G1 (30%), AES (35.14%), GLDA (31.63%) and GG1 (33.83%). A low relative abundance of the Acidobacteria phylum was detected in CK (6.64%), while higher relative abundances were detected in the other five treatments: G1 (12.14%), AES (12.09%), GLDA (13.47%), AG1 (13.31%) and GG1 (16.26%). The Chloroflexi phylum showed a similar growth trend. This implies selective enrichment of Acidobacteria and Chloroflexi. This phenomenon may be due to the fact that these two phyla are known to be related to metal reduction [40]. In the CK treatments, a higher relative abundance (26.64%) of the Actinobacteria phylum was detected, while lower relative abundances were detected in the other five treatments: G1 (25.05%), AES (22.44%), GLDA (24.02%), AG1 (18.65%) and GG1 (22.59%). Meanwhile, Firmicutes and Bacteroidetes showed a similar decreasing trend.

To better identify key taxonomic groups affected by GA_3_, AES and GLDA, we classified the soil bacteria to the genus level (figure 5*b*). The plant extraction efficiency is directly related to the effectiveness of phytoremediation of soil heavy metal pollution. Among the treatments, the highest extraction amount and extraction efficiency of the plants were achieved with the AG1 treatment. A review of the literature revealed that some dominant genera in the soil samples under the AG1 treatment have been reported as tolerant to heavy metal stress. The main dominant genera (top 5) in the AG1 group were *Aminobacter* (22.17%), *norank_c__Subgroup_6* (8.14%), *Sphingomonas* (5.09%), *norank_c__KD4-96* (3.60%) and *norank_f__JG30-KF-CM45* (2.53%).

### 3.5. Redundancy analysis of soil physicochemical properties and soil microbial communities

**Figure 6.**
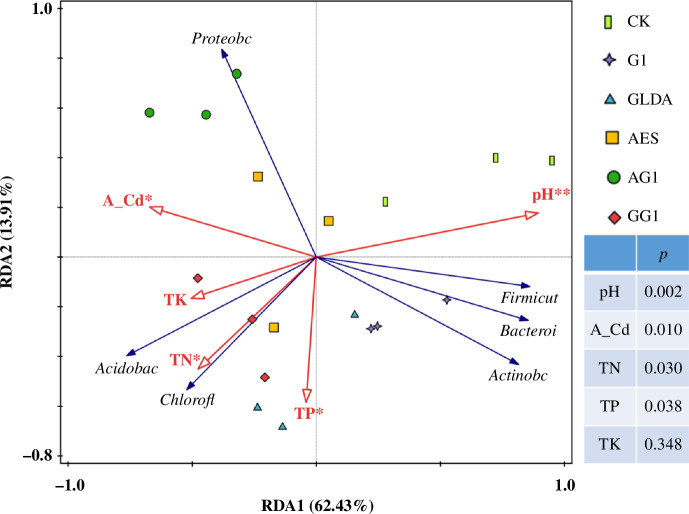
RDA analysis of dominant strains and environmental factors. Note: red rays represent soil environment variables, blue rays represent species variables. A_Cd is an abbreviation for available Cd. *Significant correlation (*p <* 0.05) and **highly significant correlation (*p <* 0.01).

**Figure 7.**
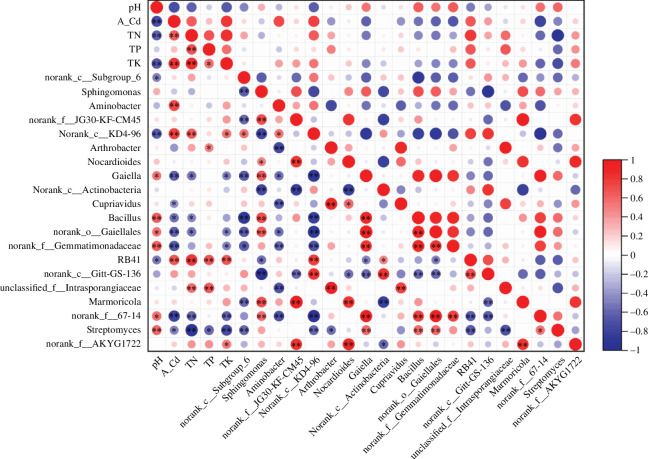
Heat map of correlation between the top 20 colonies at genus level of soil microorganisms and soil physical and chemical properties. Note: indicators on the axes represent soil environmental factors and bacterial species, and the scale on the right-hand side represents the *R* values, which are indicated by different colours in the figure. *Significant correlation (*p <* 0.05) and **highly significant correlation (*p <* 0.01).

The application of chelating agents and plant growth regulator affects soil physicochemical properties, and changes in these properties have been recognized as a key factor affecting the microbial community structure [41]. Our study shows that soil physicochemical properties correlate with the abundance and diversity of the soil bacterial community. In order to elucidate the interactions between changes in soil physical and chemical properties and soil microbial community structure, RDA was used to assess the relationship between bacterial soil microbial communities at the phylum level and soil physicochemical properties (figure 6). In addition, Spearman analysis was used to assess the relationship between soil physicochemical property parameters, Cd concentration and relative abundance of dominant bacterial phyla and genera under AG1 treatment (figure 7).

As shown in figure 6, the red ray represents the soil environment variable, the blue ray represents the species variable and the cosine value of the angle between any two arrows indicates the magnitude of the correlation between these two influence factors. If the angle is acute, the correlation is positive; the smaller the angle, the higher the correlation and greater than 90°, the correlation is negative. The eigenvalues of the first axis explained 62.43% of the variation in community composition; the eigenvalues of the second axis explained 13.91%; and the two cumulatively explained a total of 76.34% of the information.

The RDA results indicate that soil pH is an important factor affecting the composition of the bacterial community, followed by soil available Cd, TP, TN, and TK also playing a key role in the composition of microbial communities (figure 6). The present study showed that soil physicochemical properties were correlated with soil microbial community abundance and diversity, and the application of GA_3_ and chelator AES affected soil physicochemical properties such as soil pH, soil available Cd concentration and TP content, which in turn affected the soil microbial community. Soil microorganisms play a very important role in nutrient cycling in soil ecosystems and can be used as a sensitivity indicator of soil ecological functions during remediation of heavy metal-contaminated soils [36,37].

In this study, TN showed a significant positive correlation (*p <* 0.05) with the abundance of Chloroflexi and Acidobacteria (figure 6). It was further observed that *norank_c__KD4-96* and *norank_f__JG30-KF-CM45*, of the Chloroflexi phylum, were positively correlated with TN, and the correlation between *norank_c__KD4-96* and TN was significant (*p <* 0.05), while the genus *norank_c__Subgroup_6*, of the Acidobacteria phylum, was positively correlated with TN (figure 6). In the present study, TP showed a highly significant positive correlation (*p <* 0.01) with the abundance of Chloroflexi, and *norank_c__KD4-96* and *norank_f__JG30-KF-CM45* (Chloroflexi) were positively correlated with TP. Conversely, TP showed a significant negative correlation (*p <* 0.05) with Proteobacteria abundance (figure 6), with *Sphingomonas* and *Aminobacter* (Proteobacteria) being negatively correlated with TP. TK showed a highly significant positive correlation (*p <* 0.01) with Chloroflexi abundance, and *norank_c__KD4-96* and *norank_f__JG30-KF-CM45* (Chloroflexi) were positively correlated with TK, with the correlation between *norank_c__KD4-96* and TK being significant (*p <* 0.05) (figure 6). Similar results were found in a related study, with the results showing that soil K elements and Chloroflexi exhibited a significant positive correlation [42].

### 3.6. Structural equation modelling between soil properties–plant indicators–soil microorganisms

**Figure 8.**
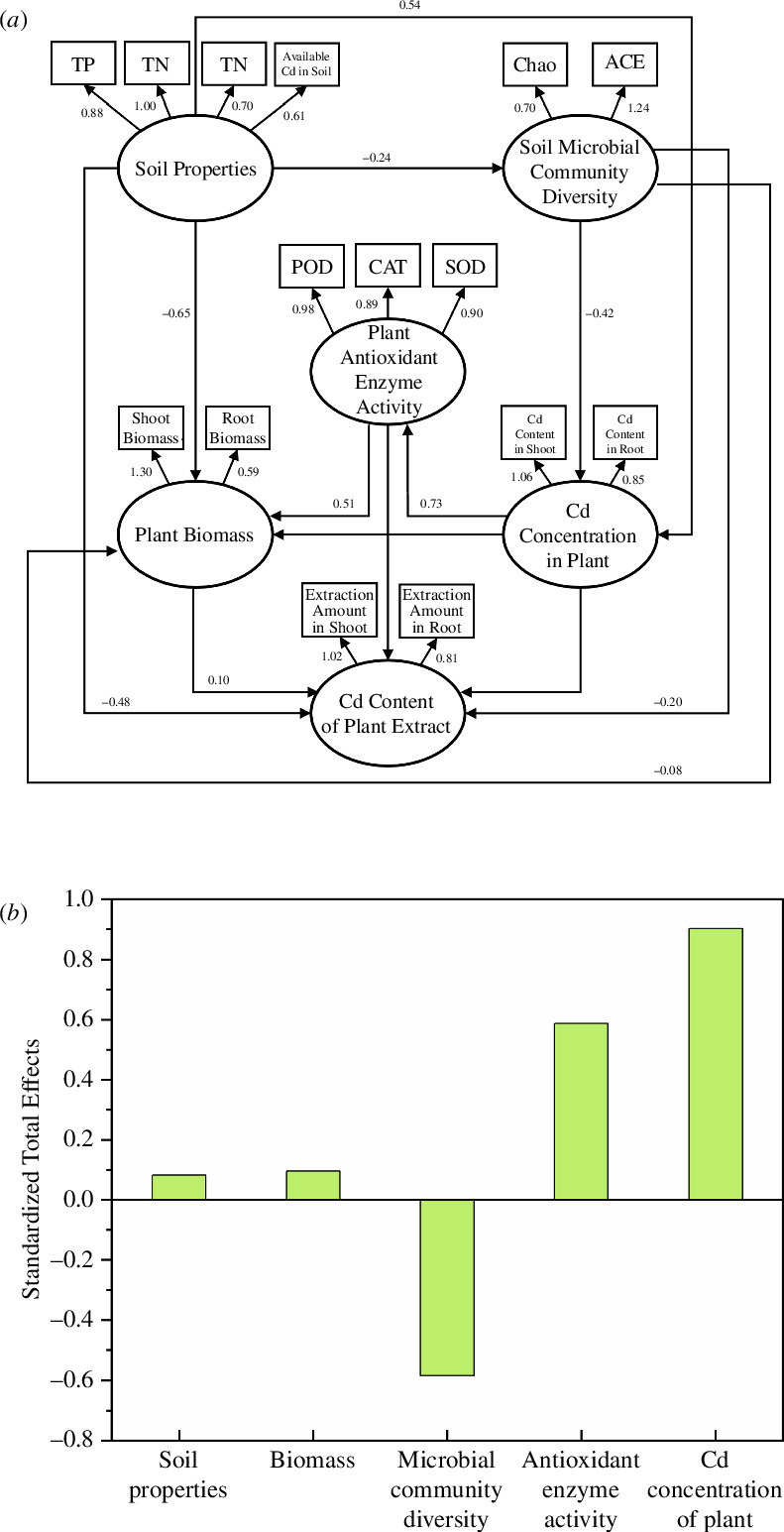
Structural equation modelling between soil–plant–soil microorganisms (*a*) and the standardized total effects obtained (*b*).

Soil properties (TN, TP and available Cd), soil microbial community diversity (Chao1 and ACE indices), plant biomass (shoot biomass and root biomass), plant Cd concentration (in shoots and roots), antioxidant enzyme activities in plants (SOD, CAT and POD activities) and plant Cd extraction (in shoots and roots) were used to construct SEM using Amos software (figure 8*a*). The SEM was constructed based on the following theoretical assumptions: changes in soil properties directly or indirectly affect plants and soil microorganisms, and all indicators have direct or indirect effects on plant Cd extraction. The SEM was drawn as shown in figure 8*a*, with elliptical boxes indicating latent variables, rectangular boxes indicating measured variables, arrows indicating correlations, arrow directions indicating the flow of causal effects and numbers next to the arrows indicating path coefficients.

The data were standardized and the model was estimated using the great likelihood estimation method, and the measures of fit were selected as the Chi-square degrees of freedom ratio (CMIN/DF) and the comparative fit index (CFI). Results show that the CMIN/DF of the structural equation was 5.302 and the CFI was 0.542. The path analysis explained 83% of the variance of the total Cd extraction from the soil by plants.

Figure 8*a* shows that each latent variable had both direct and indirect effects on plant Cd extraction. Figure 8*b* reflects the standardized total effects (direct and indirect effects) of each latent variable on plant Cd extraction. It can be seen that all latent variables, except soil microbial community diversity, have positive effects on plant Cd extraction. The highest total standardized effect was for the plant Cd concentration. In addition to the direct effect, the plant Cd concentration also indirectly affected the plant extracted Cd content through factors such as plant antioxidant enzyme activity, with a total effect of 0.900. This was followed by plant antioxidant enzyme activity, which can also indirectly affect plant Cd extraction through plant biomass, with a total effect of 0.592.

To a certain extent, the SEM constructed in this study shows the interrelationship between the ‘soil–plant–soil microorganisms’ during the remediation process was constructed in a comprehensive manner.

## 4. Discussion

### 4.1. Impact of enhanced phytoremediation techniques on maize growth

The growth of plants is a crucial factor in evaluating the efficacy of remediation techniques. The utilization of plant growth regulators can facilitate the growth and development of remediation plants, enhance the accumulation and extraction efficiency and stability of certain plants for heavy metals and consequently enhance the remediation efficiency of plants. Additionally, the plant growth regulator can also enhance the plant’s resistance by regulating the plant’s physiological and biochemical processes, thus promoting the plant’s growth [17]. It has been demonstrated that the application of GA_3_ stimulates cell division in plants and is a promoter of cell enlargement [43]. Furthermore, the application of GA_3_ can facilitate plant growth by increasing the permeability of plant cell membranes and enhancing the translocation and uptake of water and nutrients [44–47].

The results of this study indicate that the biodegradable chelating agent combined with a plant growth regulator to enhance maize remediation technology has a more pronounced effect than the control group. The biomass of maize in all treatments was increased, with the greatest increase observed in the treatment with the application of the plant growth regulator GA_3_. This was significantly higher than that of the group without the GA_3_ treatment. This is related to the enhanced scavenging ability of antioxidant enzymes by GA_3_, and the incorporation of plant growth regulators can increase the antioxidant enzyme activities in plants, significantly alleviate the membrane lipid peroxidation caused by Cd stress and reduce the MDA content in plants. Furthermore, this study found that maize biomass increased with increasing GA_3_ concentration, while it decreased when GA_3_ concentration was too high. This was due to the fact that the endogenous growth hormone content in the plant increased when the GA_3_ concentration was too high. Furthermore, the growth hormone induced ethylene production when the GA_3_ concentration exceeded the optimal concentration, which then inhibited the growth of the plant’s stem nodes and root [34,48]; alternatively, due to the toxicity of the heavy metals, the high concentration of GA_3_ promoted the growth of maize, and at the same time, the enrichment of excessive Cd resulted in the inactivation of antioxidant enzyme systems in the plant [49]. The analysis of antioxidant enzyme activity and MDA content in maize has revealed that the antioxidant enzyme system in maize is inactivated during treatment with high concentrations of plant production regulators. This results in a lower scavenging capacity for free radicals in the plant than the amount produced, which in turn exacerbates the peroxidation of the cellular lipid membranes and leads to an elevation of MDA content. This conclusion is supported by other studies. For example, the exogenous application of the plant growth regulator GA_3_ increased the activities of the antioxidant enzymes SOD, POD, CAT and APX in *Brassica juncea* L. grown in Cd- and U-contaminated soil, thereby reducing oxidative stress damage and significantly decreasing the MDA content in the plant [21].

In addition to the limitation of plant biomass, the effectiveness of phytoremediation of heavy metal-contaminated soil is also affected by the bioavailability of heavy metals. The findings of this study indicate that a low concentration of GA_3_ may mitigate the toxicity of heavy metals and facilitate the growth of maize under the AG1 synergistic treatment. This finding is consistent with a previous study that demonstrated that low concentrations (10^−6^ mol l^−1^) 0f GA_3_ and low (10^−6^ mol l^−1^) GA_3_ with EDTA significantly enhanced the growth and biomass of *Parthenium hysterophorus* L. in Cd-contaminated soil [50].

The application of GA_3_ has been demonstrated to facilitate growth and enhance the biomass of maize [51] and loblolly pine [52]. Chelating agents applied to the soil increase the availability of heavy metals in the soil and enhance heavy metal stress on plants. It has been shown that gibberellin can increase plant biomass under heavy metal stress [53,54]. By positively regulating the expression of the cellulose synthase gene CESA, gibberellin is able to control the biosynthesis of cellulose in the primary and secondary cell walls of plants and increase the cellulose content, thus preventing heavy metal ions from entering the cell protoplasts. At the same time, the increased cellulose content will also increase the length of the plant cells, resulting in their growth and expansion [55,56].

This study demonstrated that the antioxidant enzyme activity in maize was enhanced, accompanied by a significant increase in shoot biomass. The trends observed under the different treatments were found to be essentially similar. In a study of the remediation of Cd-contaminated soil with *Brassica napus* L. using citric acid, it was found that the application of citric acid increased plant biomass and reduced the MDA and H_2_O_2_ contents in the plant, thus reducing Cd stress [57].

### 4.2. Effects of intensive phytoremediation techniques on Cd enrichment in maize

Studies have demonstrated that growth regulators can regulate the transport of heavy metal ions and nutrients at the structural level within plant cells. Within the optimal concentration range, they can accelerate ion transport across the cell membrane. Consequently, the Cd concentration in shoots increased [51,58]. The manner in which plant growth regulators induce the accumulation of heavy metals in plants may be related to their promotion of the plant transpiration ratio and activation of proteins related to heavy metal transport [44,59].

The Cd content in plants treated with a chelator alone was found to be significantly higher than that in plants treated with GA_3_ alone (*p <* 0.05). However, the difference in extraction efficiency was not found to be significant. The extraction efficiency of soil heavy metals by plants is influenced not only by biomass but also by the concentration of the heavy metal in the plant. The total amount of Cd extracted by maize under AG1 treatment was maximum at 8.41 μg g^−1^ DW with an extraction efficiency of 0.52%. The AG1 treatment resulted in the highest extraction amount and extraction efficiency of the plants, indicating that while increasing maize biomass, this treatment can also significantly promote the extraction and enrichment of Cd by plants. The remediation of Cd-contaminated soil by ryegrass using GA_3_ and EDTA has been the subject of considerable research. The results of this research indicate that the remediation effect under a synergistic treatment is superior to that of treatments involving hormones and chelating agents alone. This finding is consistent with the results of the present study [60]. Under a synergistic treatment, GA_3_ can mitigate the impact of chelating agents on plants and restrict the migration of Cd in the shoots by storing Cd in the cell wall.

### 4.3. Impact of enhanced phytoremediation techniques on soil microbial communities

All treatments resulted in a reduction in the richness and diversity of soil microbial communities in comparison to CK. Among the treatments, the diversity of the soil bacterial community was reduced more by the application of chelating agents than by the hormone spraying treatment alone. This finding is consistent with the results of previous studies. When the concentration of Cd is elevated, the diversity and abundance of soil bacterial communities are diminished [61]. The AES treatment exhibited a more pronounced impact on the reduction of the bacterial community diversity than the GLDA treatment. Conversely, the application of GA_3_ on the foliar surface resulted in a greater reduction in diversity under the synergistic treatment.

In this study, the relative abundances of the Proteobacteria and Acidobacteria phyla increased under the AG1 treatment. This is likely due to the fact that Proteobacteria is the largest bacterial phylum and contains a high diversity of bacteria. The Proteobacteria phylum has been demonstrated to exhibit the greatest tolerance to heavy metals among microbial groups and is involved in nitrogen fixation and denitrification. Furthermore, it has been shown to promote plant growth [62,63]. The Acidobacteria phylum is the second most important phylum. It is self-regulating and plays an important role in soil ecosystems and in maintaining ecosystem balance [61]. In general, elevated heavy metal concentrations result in a reduction in microbial diversity, particularly among sensitive species, with resistant microbial species becoming dominant in this environment [64,65].

A significant enrichment of *Aminobacter* (22.17%) was observed in AG1, whose relative abundance group was only 0.22% in CK. *Aminobacter* has been demonstrated to possess a distinctive metabolic capacity to proliferate in ecosystems containing toxic compounds and has been shown to exhibit tolerance to Cd [66]. The high relative abundance of *Aminobacter* in the AES and AG1 treatments, in comparison to the low abundance in the other treatments, suggests that the chelator AES may be providing carbon and nitrogen as energy sources to *Aminobacter*. Furthermore, an enrichment of *norank_c__Subgroup_6* (8.14%) and *Sphingomonas* (5.09%) was observed. These genera are particularly sensitive to contamination by Cd in soil, promoting the accumulation and transport of heavy metals in plants and enhancing the phytoremediation of soil heavy metal contamination [53,67,68]. These results also elucidate the higher Cd extraction observed in AG1. Furthermore, the relative abundance of *KD4-96* was 3.60%, which was higher than that of CK (1.26%). This taxon has been reported to belong to the Chloroflexi phylum, which is known to possess a wide range of metabolic activities and ecological functions, including fermentation and anaerobic photosynthesis. These functions have been demonstrated to be positively correlated with maize growth [69]. This finding is also consistent with the results presented in §3.1 of this study. Finally, the relative abundance of *norank_f__JG30-KF-CM45* has been correlated with soil N and P concentrations, as previously reported in the literature. It is therefore possible that this bacterium may enhance organic carbon degradation related to TN and TP [70].

The results of the RDA analysis indicated a distribution of flora in which Proteobacteria, Acidobacteria and Chloroflexi were negatively correlated with soil pH and positively correlated with available Cd in the soil. This suggests that these three bacterial taxa are more resistant to heavy metals. Furthermore, N, P and K are known to be essential nutrients for organisms, while TP, TN and TK are also dominant factors in microbial biosynthesis and are involved in cellular metabolism processes, such as photosynthesis, respiration, energy storage and cell division [71,72].

The increase in TN and TK led to the enrichment of soil bacteria that promote metabolism (*norank_c__KD4-96, norank_c__Subgroup_6, norank_f__JG30-KF-CM45*), while the decrease in TP promoted the accumulation of bacteria that are tolerant to heavy metals and the transport of heavy metals in plants by promoting the enrichment of certain soil bacteria (*Sphingomonas* and *Aminobacter*), resulting in higher Cd extraction [73,74].

### 4.4. Strengthening the relationship between phytoremediation effects and environmental factors

The efficacy of intensive phytoremediation, defined as the quantity of Cd extracted from maize, is contingent upon a multitude of variables. In this article, indicators such as the nature of the soil, the growth conditions of maize and the concentration of Cd in its body were selected. Based on reasonable assumptions, a SEM was calculated and plotted. The results indicate that the concentration of Cd and the activity of antioxidant enzymes in maize were the primary influencing factors. All of these factors exhibited a positive effect, as did maize biomass and soil properties. The concentration of Cd in maize and the biomass of the plant were the most direct factors affecting the amount of Cd extracted from maize. In contrast to hyper-enriched plants, the advantage of a large biomass of enriched plants meant that their remediation effect was better. Furthermore, the concentration of Cd in maize indirectly affected the amount of Cd extracted from maize by changing the activity of antioxidant enzymes. Finally, the concentration in maize showed a positive effect on the antioxidant enzyme activity. In response to unfavourable growth conditions, plants are able to adopt multiple defence mechanisms to repair the damage caused by ROS. This process is accompanied by an enhancement of antioxidant enzyme activities [75]. It has been demonstrated that the augmentation of antioxidant enzyme activities, including those of POD, SOD and CAT, constitutes a pivotal material basis for the enhancement of plant tolerance to heavy metal stress [76,77]. In the present study, antioxidant enzyme activities were significantly increased in all treatments compared to the control, indicating that maize was experiencing enhanced stress due to Cd exposure.

It has been demonstrated that soil microorganisms are more sensitive to heavy metal contamination and also influence the transport of Cd in soil [78]. Soil microorganisms may alter the modes of heavy metals, such as dissolution and leaching, which in turn affects the accumulation of heavy metals in plants [79,80]. The Chao and ACE indices, which reflect changes in microbial community richness, demonstrated a negative impact on corn Cd extraction. This indicates that a decline in soil microbial community richness will result in an increase in corn Cd extraction. The reason for this may be that an increase in the concentration of Cd in the soil leads to an increase in corn Cd extraction. However, at the same time, the soil microbial community was greatly affected by Cd, with a concomitant decrease in richness and an enrichment of dominant Cd-resistant bacteria. This is confirmed by the analysis of soil microbial communities presented in §3.4. Some scholars have conducted similar structural equation modelling studies, and the results indicated that the pathway of soil microbial activity–effective state heavy metal content–plant heavy metal accumulation was a negative coefficient. In this pathway, the effective state heavy metal content played an intermediary role in the influence pathway [81]. In this article, we consider the effective state heavy metal content to be generalized to the soil properties, and we obtained results that were essentially the same. An increase in the diversity of soil microbial communities is likely to have a detrimental effect on the accumulation of heavy metals in plants. This is because an enhanced rate of soil respiration may favour the bioconcentration of heavy metals by soil microbes, thereby inhibiting the uptake of heavy metals by plants [82].

## 5. Conclusion

Cd is the primary heavy metal pollutant affecting the environmental quality of soil in agricultural land, which seriously threatens food security and human health. The search for cost-effective and environment-friendly remediation technologies is urgent. In this study, we constructed an intensive phytoremediation technology, which is to remediate Cd-contaminated soil by enhancing maize with biodegradable chelating agents (AES and GLDA) and plant growth regulator (GA_3_). We investigated the remediation effect and mechanism of this technology, analysed the effects of the remediation process on the abundance and diversity of soil microbial communities and explored the correlations between soil physicochemical properties and microbial communities, environmental variables and plant extraction of Cd. The main conclusions are as follows. (i) The treatments of 10^−6^ mol l^−1^ GA_3_ and 6 mmol kg^−1^ AES-enhanced maize were more effective in remediation of Cd-contaminated soil, with the total biomass and extraction efficiency of maize reaching 3.6 and 8.67 times those of the control, respectively. The remediation technology promoted the increase of antioxidant enzyme activities and biomass in maize, which in turn enhanced the extraction of Cd by the plant. (ii) Under AES combined with GA_3_ treatment, the dominant genera that promote plant growth and metabolism and strong resistance to heavy metal stress were enriched, which in turn promoted the growth of maize and the enrichment of Cd. (iii) The results of structural equation modelling indicated that the effects of Cd concentration in plants and plant antioxidant enzyme activities on plant Cd extraction were large.

Green and efficient intensive phytoremediation is the future development direction to achieve the remediation of heavy metal contamination in agricultural land, and the results of this study show that enhanced phytoremediation technology has a good potential to be applied in the remediation of Cd-contaminated soil. The research results provide a theoretical basis and technical support for the remediation of Cd-contaminated agricultural soils and also have important positive significance for the safe use and scientific management of heavy metal-contaminated agricultural soils. In subsequent research, for the Cd-contaminated soil in different regions, the combination of different plants with degradable chelating agents and plant growth regulators can be explored according to local conditions, so as to enrich the selection and application scope of soil pollution remediation technology.

## Data Availability

Additional data are available at Dryad [83].
